# Prognostic Impact of Modulators of G proteins in Circulating Tumor Cells from Patients with Metastatic Colorectal Cancer

**DOI:** 10.1038/srep22112

**Published:** 2016-02-26

**Authors:** Jorge Barbazan, Ying Dunkel, Hongying Li, Ulrich Nitsche, Klaus-Peter Janssen, Karen Messer, Pradipta Ghosh

**Affiliations:** 1 Translational Laboratory, Medical Oncology Department, Complexo Hospitalario Universitario de Santiago de Compostela/SERGAS, Santiago de Compostela, Spain; 2 Department of Medicine, University of California, San Diego, La Jolla, California, USA; 3 Department of Surgery, Klinikum rechts der Isar, TU München, Munich, Germany; 4 Division of Biostatistics, Department of Family Medicine and Public Health, Moores Cancer Center, University of California, San Diego, La Jolla, California, USA

## Abstract

The consequence of a loss of balance between G-protein activation and deactivation in cancers has been interrogated by studying infrequently occurring mutants of trimeric G-protein α-subunits and GPCRs. Prior studies on members of a newly identified family of non-receptor guanine nucleotide exchange factors (GEFs), GIV/Girdin, Daple, NUCB1 and NUCB2 have revealed that GPCR-independent hyperactivation of trimeric G proteins can fuel metastatic progression in a variety of cancers. Here we report that elevated expression of each GEF in circulating tumor cells (CTCs) isolated from the peripheral circulation of patients with metastatic colorectal cancer is associated with a shorter progression-free survival (PFS). The GEFs were stronger prognostic markers than two other markers of cancer progression, S100A4 and MACC1, and clustering of all GEFs together improved the prognostic accuracy of the individual family members; PFS was significantly lower in the high-GEFs versus the low-GEFs groups [H.R = 5, 20 (95% CI; 2,15–12,57)]. Because nucleotide exchange is the rate-limiting step in cyclical activation of G-proteins, the poor prognosis conferred by these GEFs in CTCs implies that hyperactivation of G-protein signaling by these GEFs is an important event during metastatic progression, and may be more frequently encountered than mutations in G-proteins and/or GPCRs.

Heterotrimeric G proteins and G-protein-coupled receptors (GPCRs), which comprise the largest family of signaling hubs in eukaryotes, have long been recognized as crucial players in tumor growth and metastasis (reviewed in^1^,^2^). Cancer cells often hijack the G-protein/GPCR signaling pathway to orchestrate advantageous phenotypes at various stages of oncogenic progression, e.g., neoplastic transformation, survival, proliferation, immune evasion, angiogenesis, and invasion into surrounding tissues to spread to distant organs. Multiple studies examining rare oncogenic driver mutations in G proteins or their modulators [summarized in^1^,^2^] have established that “*hyperactivation of G proteins*” is a *bona-fide* basis for oncogenic signaling via trimeric G proteins. However, these rare mutations do not explain the basis for deregulated G protein signaling in the vast majority of cancers. A growing body of work by us and others^3^,^4^,^5^,^6^ has defined a more frequent alternative mechanism by which cancer cells may hijack G protein signaling pathways and in this way fine tune to their advantage signaling networks that are triggered by growth factors, extracellular matrix, and other ligands. This alternative mechanism is a non-canonical mode of activation of G proteins that is not initiated by GPCRs, but instead by a recently identified family of non-receptor GEFs, called rheostats^7^. Rheostats, including Gα-Interacting Vesicle-associated protein (GIV; a.k.a Girdin)^3^,^4^ and the 3 other family members Daple^8^, Calnuc/NUCB1 and NUCB2^9^, serve as GEFs for the inhibitory G protein α-subunit, Giα, via an evolutionarily conserved motif (Fig. 1). The name rheostat was chosen to indicate the ability of cells to ‘adjust’ the duration and extent of G protein signaling by altering the abundance of functional copies of these GEFs in cells^7^. While the molecular mechanisms that govern this non-canonical G protein activation and the variety of pathways or pathophysiologic processes they modulate are still unfolding [summarized in^3^], the relevance of this new paradigm in cancer progression is clear [summarized in^4^; Table S1]. Although each of the four members of the GEF family has a distinct molecular make-up (Fig. 1), different subcellular localization, and a preferred set of receptors that they target and signaling pathways that they modulate, each has been linked to cancer cell migration and/or invasion across a variety of cancers (Table 1). Importantly, increased expression of these non-canonical GEFs in primary tumors has been associated with increased risk of metastatic progression and/or poor clinical outcome (multiple citations, Table 1).

Despite the insights gained in pro-tumorigenic/pro-metastatic roles of each member of this family, and the prognostic significance of individual members, the significance of elevated expression of all members combined has not been studied. Here we evaluated the prognostic significance of individual members of this new family of modulators of G protein, and analyzed the combined predictive power of all members of this family.

## Results and Discussion

### Expression of GEFs is increased in the invasive edge of primary colon tumors and in metastatic tumors compared to the non-invasive core

**Figure 2:**
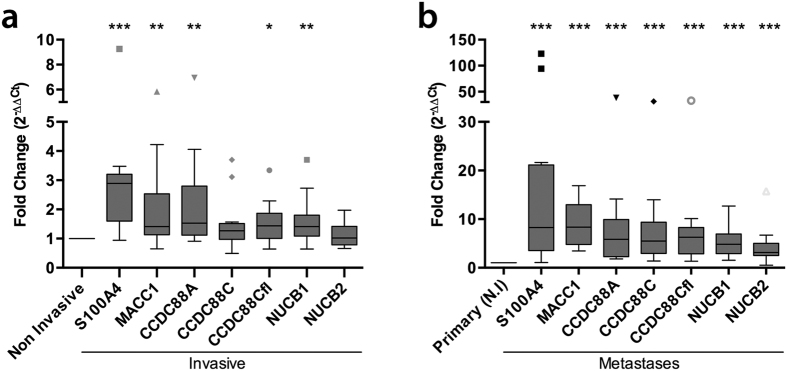
Comparison of levels of mRNA expression of selected markers in invasive vs non-invasive edges of primary tumors (**a**) and primary vs metastatic CRC tissue (**b**). (**a**) Levels of mRNA expression of a panel of genes was analyzed in the invasive front and the corresponding non-invasive central areas of the same tumor (n = 13) by qPCR. Box plots show the fold change in levels (Y axis) of expression normalized to non-invasive tumor tissue. The statistical significance of the differences for individual genes in both tumor areas was calculated applying a non-parametric Wilcox signed rank test. (**b**) Levels of mRNA expression of a panel of genes was analyzed in an independent set of metastatic tissue (n = 14, 7 lung metastases and 7 liver metastases) and compared to the mean levels of expression of each gene in the non-invasive (N.I) area of primary colorectal tumors. Box plots display the fold change in levels (Y axis) of expression normalized to non-invasive tumor tissue. Statistical significance was analyzed as in A. Multiple comparison adjustment was performed. *p < 0,05; **p < 0,01; ***p < 0,001.

We chose to study the combined prognostic significance of GEF family members in colorectal cancer (CRC) because that is the type of cancer where the prognostic significance of each member of the GEF family has been studied individually (Table 1). First we analyzed the relative mRNA expression of each GEF, GIV (CCDC88A), Daple (CCDC88C and CCDC88Cfl), NUCB1 and NUCB2 in the invasive edge and the non-invasive center of primary tumors (Fig. 2a). All probes were designed to specifically analyze the isoforms that contain the GEF module. In the case of Daple, two GEF-containing isoforms have been reported in the National Center for Biotechnology Information (NCBI) database, a full-length reference sequence form and a shorter form that lacks the N-terminus. Thus, we used two different probes for Daple: one that amplifies a region within the C-terminus which contains the GEF motif (CCDC88C) and one that amplifies a region within the N-terminus (CCDC88Cfl; can only recognize the full length form). In the absence of *bona-fide* CTC biomarkers in CRC, as positive controls we analyzed two established markers of aggressiveness and metastatic potential, S100A4 [S100 Calcium Binding Protein A4^10^,^11^,^12^,^13^,^14^,^15^;] and MACC1 [metastasis associated in colon cancer 1^16^,^17^;]. All 4 members of the GEF family were expressed at significantly higher levels at the invasive edge compared to non-invasive core; the fold increase was highest in the case of GIV/CCDC88A (Fig. 2a). As anticipated, both MACC1 and S100A4 showed elevated expression in invasive edges compared to non-invasive cores of the same tumor.

Next, we asked how the expression level of each GEF family member changed during metastatic progression. We analyzed the abundance of mRNA in 14 metastatic tumor samples (7 from liver and 7 from lung) and compared these to mRNA levels in the non-invasive central cores of 13 primary tumors. Expression levels of all genes, i.e., GEFs and positive controls, S100A4 and MACC1 were elevated in metastatic lesions compared to primary tumors; the fold increase was highest in the case of S100A4 (Fig. 2b). These findings confirmed the previously defined roles of MACC1 and S100A4 in metastasis, and provided evidence for the involvement of GEF-related genes in CRC progression. These findings also underscore the limitations of biomarker studies, i.e., primary and metastatic tumors are composed by a variety of different cellular subtypes that confer them high degree of heterogeneity; such heterogeneity is spatially (non-invasive core vs invasive periphery) and temporarily variable and altered by the administration of anticancer drugs^18^.

### Expression of GEFs is increased in CTCs from patients with metastatic CRC compared to healthy volunteers

**Figure 3:**
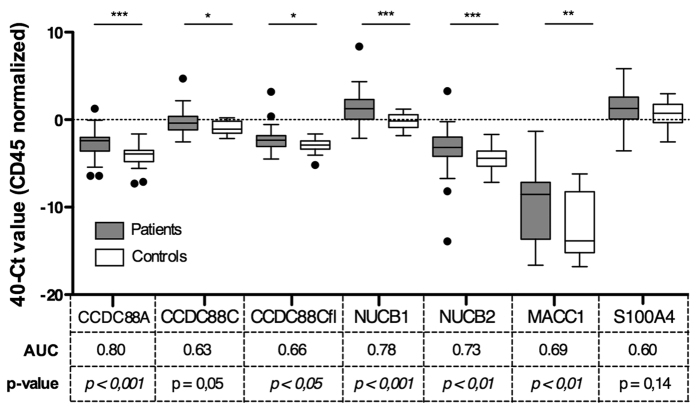
mRNA expression levels for selected genes in CTCs enriched from the peripheral blood of patients with metastatic colorectal cancer (mCRC) and healthy controls. CTC-enriched samples from the peripheral blood of 51 patients with mCRC and from 24 healthy volunteers were analyzed for mRNA levels for selected genes. Box plots show relative Ct values for mRNA from patients (gray boxes) and healthy controls (white boxes). The y axis is the qPCR Ct value for each gene, subtracted from 40 (max Ct value possible), and normalized to CD45 Ct count. CD45 is a commonly used marker of non-specific contamination with human peripheral blood lymphocytes^30^; CD45 level did not differ significantly between patients and controls. Area under the ROC curve (AUC) is presented below each marker, along with the p values (see *Methods*). *p < 0,05; **p < 0,01; ***p < 0,001.

To overcome the limitations of analyzing tissue samples from primary tumors, e.g., sampling biases arising from tumor heterogeneity, restricted number of biopsies, quantity and location^19^, we chose to study circulating tumor cells (CTCs). CTCs are believed to be central players in tumor dissemination^20^,^21^,^22^,^23^,^24^. Despite their heterogeneity and low frequency of appearance in circulation^25^, the ability to analyze CTCs has been likened to ‘liquid biopsy’ for prognostication and prediction, allowing repeated temporal access and spatial sampling of the whole tumor^26^,^27^,^28^,^29^,^30^. To investigate whether elevated expression of GEFs in CTCs might be used as a prognostic measure, we compared GEF expression in EpCAM-isolated CTCs from the peripheral blood of 51 patients with metastatic CRC (Table 2) to similarly treated samples from 24 healthy donors. For each of the 4 GEFs, expression level was higher in the patient, samples compared to the healthy donors (Fig. 3), with GIV (CCDC88A), NUCB1 and NUCB2 displaying the highest differences in expression. We assessed the ability of each gene to discriminate between patients and controls using area under the receiver operating characteristic curves (AUC). These analyses showed that CCDC88A was better able to discriminate patients and controls than the other genes analyzed (AUC = 0,80, p < 0,001; Fig. 3). Considering the known prognostic markers S100A4 and MACC1, we found a significantly higher expression of MACC1 (p < 0,001) in samples obtained from patient with metastatic CRC compared to controls. The expression level of S100A4 showed a similar trend, but was not significant (p = 0,14). These results indicate that the background levels of mRNA of various members of the GEF family in the peripheral blood of healthy volunteers is relatively low, and may potentially serve as useful tools in CTC detection in the peripheral blood of patients afflicted with colorectal cancer.

### High expression of GEFs in CTCs is associated with shorter progression-free survival

Next we investigated whether the expression level of GEFs in the CTCs was associated with disease progression or survival. We constructed Kaplan-Meier survival curves for both progression-free survival (PFS) and overall survival (OS) for each marker. Patients were grouped into “high” or “low” expression groups depending on whether the level of expression was above or below the 75% percentile cutoff value for each independent marker, as previously shown^31^. All GEFs showed significant prognostic value for PFS (Table 3, left columns); the median time to progression was significantly shorter in patients with high expression in CTCs compared to patients with low expression. NUCB1 had the strongest association with PFS; median PFS was twice as long in patients with low levels of NUCB1 compared to those with high levels of NUCB1 (10,6 vs 5,2 mon, p < 0,001) (Table 3). In the case of OS, for all GEFs the median time to death was shorter in patients with high expression compared to those with low expression (Table 3, right columns), however only Daple (CCDC88C and CCDC88Cfl) and NUCB1 reached statistical significance (Table 3, right panel). In the case of our positive controls, MACC1 and S100A4, although high expression was associated with shorter survival, surprisingly, only S100A4 was significantly associated with PFS, and neither was significant for OS (Table 3).

We used univariate Cox regression to compare the potential prognostic performance of the GEFs with that of the standard clinical parameters (Table 4). Among clinical parameters analyzed, only the presence or absence of lung metastases had a significant impact on both PFS and OS (Table 4), whereas the number of metastatic sites (≤2 vs >2) and ECOG performance status were significantly associated with OS alone (Table 4). Consistent with prior findings^27^, the serum levels of Carcinoembryonic Antigen (CEA) failed to show an association with survival. By contrast, each member of the GEF family showed a strong and significant association with PFS, with Hazard Ratios (HR) ranging from 2.51 for GIV(CCDC88A) to 3.62 for NUCB1 (Table 4). Consistent with the Kaplan-Meier analyses, only Daple (CCDC88C) and NUCB1 were significantly associated with OS, with HR of 2.88 for Daple and 3.01 for NUCB1. The HRs for MACC1 and S100A4 were smaller than for the GEF family members, not reaching statistical significance in most cases (Table 4). Taken together, these results demonstrate the potential of individual members of the GEF family to be prognostic tools in CTCs, as high expression of each GEF conveyed a significantly worse prognosis.

### Grouping related genes into clusters improves the prognostic value of GEFs

**Figure 4:**
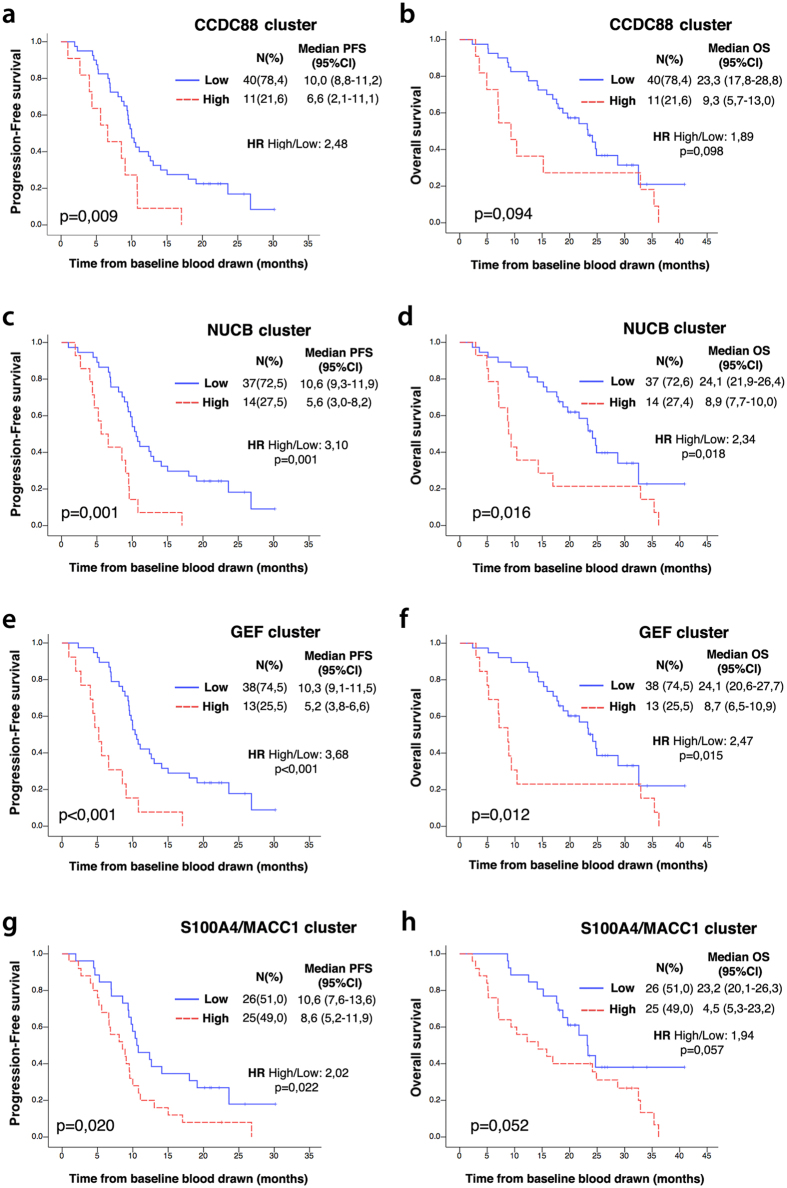
Association of progression-free (PFS) and overall (OS) survival with gene cluster expression level. Kaplan-Meier plots for PFS (left) and OS (right) are shown for dichotomized expression levels of CCDC88 cluster (CCDC88A and CCDC88C) **(a,b)**, NUCB cluster (NUCB1 and NUCB2) **(c,d)**, all GEF cluster (CCDC88A, CCDC88C, NUCB1 and NUCB2) **(e,f)**, and the positive control, S100/MACC1 cluster **(g,h)**. Red, expression above the 75^th^ percentile; Blue, expression below this level (see text for details). N = number of patients in each group. H.R = Hazard ratio. Median PFS and OS are expressed in months. *CI*: Confidence interval.

Because each member of the GEF family showed prognostic value individually, we asked if different combinations of GEFs, i.e., gene clusters, might improve the prognostic strengths of individual markers alone. Clusters were defined based on the degree of similarity between each member, e.g., the CCDC88 cluster included the two closely related orthologues GIV and Daple (CCDC88A, CCDC88C and CCDC88Cfl) and the NUCB cluster included the two closely related orthologues NUCB1/Calnuc and NUCB2. Patients were classified as high/low CCDC88 when 2 of the 3 CCDC88A/C probes were in agreement that levels of GIV and/or Daple were above/below the previously chosen cutoff. For the NUCB cluster, patients were classified as high when either NUCB1 or NUCB2 were above the previously chosen cutoff. No improvement in prognostic power was seen in either the CCDC88 or NUCB clusters when compared to individual markers alone, both for Kaplan-Meier and Cox survival analyses (Fig. 4a–d). However, when all GEFs were clustered together (CCDC88A, CCDC88C, CCDC88Cfl, NUCB1 and NUCB2), classifying patients as high GEF expression when at least 3 markers were expressed at levels higher than cutoff, we could see an improvement in prediction of PFS (Fig. 4e,f). The median PFS was 10.3 mon among patients classified as low GEF, whereas the median PFS was reduced by half, i.e., 5.2 mon among those patients classified as high GEF [HR of 3.68 (p < 0,001)] (Fig. 4e,f). Of note, clustering of the two unrelated genes, S100A4 and MACC1, our two positive controls did not show any improvement of prognostic power, and this cluster continued to perform poorly compared to the GEFs (Fig. 3g,h).

**Figure 5:**
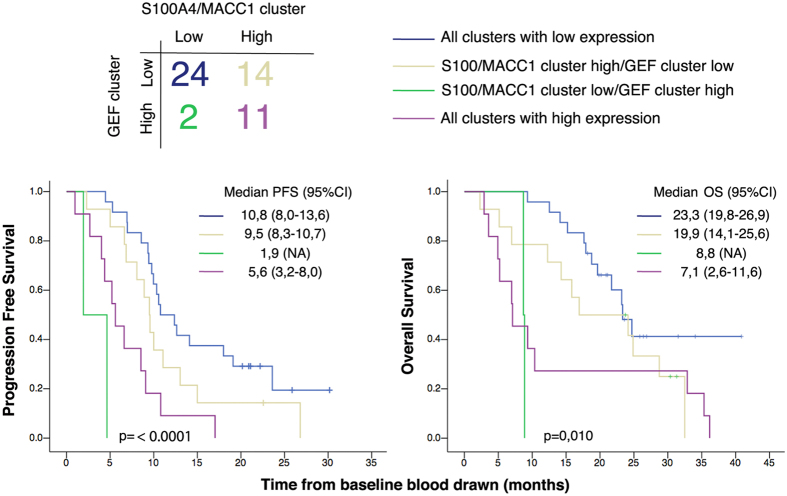
Stratification by GEF cluster expression level further improves the prognostic power of MACC1/S100A4. *Top*: Patients were classified into four groups, by expression levels of the GEF cluster or S100A4/MACC1 cluster. *Bottom*: Kaplan-Meier plots of PFS and OS for each group. Median PFS and OS are expressed in months. *CI*: Confidence Interval. Pair wise comparison of groups 1 through 4 against each other for progression free and overall survival is tabulated in Table S3.

Finally, we asked if the GEF and the S100A4/MACC1 clusters might show additive value in accurately classifying patients into good and poor prognosis groups. Patients were classified into four groups depending on the expression levels (low or high) of GEF and S100A4/MACC1 clusters (Fig. 5, upper panel). Patients with low expression levels for both clusters (n = 24) had the best PFS and OS among all the patients (Fig. 5, lower panel; blue line). By contrast, patients with high expression in CTCs for both clusters (n = 11) had lower PFS and OS (Fig. 5, lower panel; magenta line). We found that in ~30% (16/51) of patients there was no agreement between the GEF cluster and S100A4/MACC1 cluster; 14 patients had high expression of S100A4/MACC1 cluster but low expression of GEF cluster (Fig. 5, yellow line), whereas 2 patients high GEF expression but low levels of expression of S100A4/MACC1 (Fig. 5, green line). A Kaplan-Meier analysis confirmed that the presence of high levels of GEF was an overriding prognostic factor despite low levels of S100A4/MACC1 both for PFS as well as OS, i.e., the patients with high-GEF-low-S100A4/MACC1 signature lived shorter (Fig. 5, green line) than the patients with low-GEF-high-S100A4/MACC1 signature (Fig. 5, yellow line). Taken together, these findings indicate that the GEF cluster is strongly associated with survival, suggests that it adds significant information above the currently available markers S100A4 and MACC1.

To investigate the independent prognostic value of the GEF cluster, we used multivariate Cox regression (Table 5). In addition to the GEF and S100A4/MACC1 clusters, we included the three clinical variables that previously showed a significant univariate association: 1) the presence of lung metastases, 2) ECOG performance status, and 3) the number of metastases (Table 4). In this multivariate model, the GEF cluster remained an independent significant prognostic factor [HR: 5,20, p < 0,001; Table 5] for PFS, after adjusting for the effects of the clinical variables and S100A4/MACC1 cluster. However, the S100A4/MACC1 cluster was no longer statistically significant (likelihood ratio test p = 0.86). Removing S100A4/MACC1. improved the prognostic strength of the model [concordance (c)-index = 0.71 instead of 0.69, one-sided p = 0.048]. By contrast, the c-index from the model including the GEF cluster and the clinical variables was significantly higher than from the model with the clinical variables only [c-index 0.71 instead of 0.63, one-sided p = 0.007]. These findings demonstrate the added prognostic value of the GEF-cluster for PFS, over and above clinical variables. In the case of OS, the number of metastases continued to show the strongest effect on survival [HR: 4,38; p = 0,0140] and the GEF-cluster continued to be a significant prognostic factor [HR: 2,69; p = 0,031], whereas the S100A4/MACC1 cluster failed to reach statistical significance.

## Conclusions

The major finding in this work is the demonstration of the individual and combined prognostic impact of members of a new family of GEFs in CTCs isolated from patients with advanced CRC. The usefulness of CTCs as a direct indicator of patient prognosis and therapy response has gained traction in recent years, with incorporation of CTC enumeration as a parameter to guide treatment plans in the clinical setting for colon^28^,^29^, prostate^32^ and breast^33^,^34^ cancers. There has even been speculation that CTC evaluation may potentially become a test in oncology that is on a par with blood glucose measurements in diabetics^35^.

Despite this progress, there is substantial agreement that analysis should incorporate molecular profiling of CTCs, not just enumeration, in order to assess their metastatic potential and predict either tumor progression, detect relapse, or monitor response to specific therapies^35^. Prior studies have demonstrated the ability of a panel of markers to improve the overall prognostic impact, compared to individual targets^31^,^45^,^46^,^47^,^48^,^49^, and the ability of molecular profiling of CTCs from different tumor types, for gene expression as well as mutational status of key cancer-related genes (KRAS^36^, BRAF^37^, PI3KCA^38^,^39^, EGFR^40^, etc) to provide valuable insights into the biology and behavior of the primary tumor^41^,^42^,^43^,^44^. In the present study, clustering of GEFs together improved the prognostic accuracy of the individual family members. Surprisingly, the GEFs fared better as prognostic markers than two established markers of cancer progression, S100A4 and MACC1. The fact that MACC1 did not demonstrate a significant prognostic effect in our analysis could suggest that the prognostic/predictive impact of MACC1 is limited to cell-free RNA in the peripheral circulation, as shown previously^16^.

Another insight gained from this work is that increased expression of each of the 4 known members of this family is individually associated with poor outcome. Because the only shared module of all 4 molecules is a G protein regulatory motif which exerts GEF activity, it is possible that their elevated expression may synergistically contribute to and serve as a surrogate measure of elevated G protein activation during cancer progression. Based on the fact that all the members of the GEF family are widely relevant in the metastatic progression of a variety of cancers [summarized in Tables 1 and S1], we speculate that the prognostic utility of this panel of markers in CTCs will also be useful in other cancers beyond CRCs.

The current study also has implications for understanding G protein biology. The contribution of hyperactivated G protein signaling in cancers is currently interrogated using a genomics approach to identify and investigate infrequent oncogenic mutations in G proteins and GPCRs in the primary tumor tissue. One major limitation of such approach is that it ignores the impact of deregulated expression of genes other than GPCRs which coordinately function within the G protein regulatory network to maintain finiteness of G protein signaling, e.g., non-receptor GEFs which can also activate G proteins in a GPCR-independent manner, GTPase accelerating proteins (GAPs) which terminate G protein signaling, and guanine nucleotide dissociation inhibitors (GDIs) which maintain G proteins in an inactive GDP-bound state. The current study, which evaluated an entire family of non-receptor GEFs shows that aberrant expression of these network of regulatory proteins may contribute to hyperactivation of G proteins relatively more frequently than mutations in G proteins/GPCRs. By revealing the prognostic impact of elevated expression of individual as well as clusters of non-receptor GEFs on survival, this work reveals the benefit of transcriptome analysis of G protein regulatory proteins in cancer biology.

Our study has several limitations. While EpCAM, also known as HEA or BerEP4, is one of the most commonly used markers for positive isolation and detection of CTCs from patient blood its use has limitations. The occurrence of EMT in tumor cells lead to downregulation of epithelial markers including EpCAM and reduces the sensitivity for detection of CTC^50^. However, it has been demonstrated that at least a subpopulation of CTCs might reflect a partial mesenchymal phenotype, in that, they express both epithelial markers (like EpCAM) and mesenchymal markers (that are upregulated during EMT) simultaneously^35^. It is perhaps because of this reason that several studies have shown that EMT markers are indeed detected in EpCAM-isolated CTCs and have prognostic value (reviewed in Bednarz-Knoll N *et al.*, Cancer Metastasis Reviews, 2012). Furthermore, because increased expression of all 4 GEFs, i.e., GIV, Daple and Calnuc/NUCB2 are all associated with increased invasiveness and/or EMT-like phenotype [see Table S1], it is likely that the use of EpCAM to isolate CTCs may result in a significant underestimation of the abundance of CTCs that overexpress one or more of these GEFs and display EMT. Another limitation of this study is a relatively small cohort of patients in this study from a single center. Multicentric trials on larger cohorts, using either the same analysis methodology or, incorporating them to existing technologies are essential to fully realize the potential of these markers.

## Methods

### Gene expression analysis in primary tumors and metastatic tissue

Primary colorectal carcinomas (n = 13) and metastasis (liver metastasis, n = 7; lung metastases, n = 7) were processed by the Tissue Biobank, Pathology Department, Complexo Hospitalario Universitario of Santiago de Compostela. Non-invasive and invasive areas of primary tumors were identified by H&E staining and macroscopically dissected by an experienced pathologist, ensuring similar tumor cell percentages. RNA was purified (TRIZOL reagent, Invitrogen; RNeasy kit, Qiagen), cDNA was synthesized (MuLV reverse transcriptase, Life Technologies), and gene expression was evaluated using hydrolysis probes (Life Technologies) (see Table S2 for probe details). Data was represented as fold change relative to the expression in the non-invasive area. *GAPDH*, *ACTB* and *RLPLO* were used as reference genes.

### CTC Study design

51 patients with metastatic CRC were recruited at the University Clinical Hospital of Santiago de Compostela (Spain) (Table 2). Inclusion criteria were the presence of measurable metastatic colorectal cancer (stage IV), a Performance Status (PS) equal or less than 2, based on the Eastern Oncology Cooperative Group (ECOG) scale, and the initiation of a first systemic chemotherapy line. Patients treated with fluoropyrimidines (fluorouracil or capecitabine) alone or in combination with oxaliplatin/irinotecan and biological targeted therapies (Bevacizumab, Cetuximab) were included. Patients reporting previous cancer episodes treated or not with chemotherapy, were excluded from the study. The evaluation of tumor burden, metastasis location and therapy response were evaluated by standard imaging procedures (computed tomography, CT) by a specialized radiologist. Following RECIST 1.1 guidelines^51^, disease progression was defined as an increase in the number of metastatic lesions, growth of preexisting distant tumors in more than 20% of the initial size, or both. Patients who died during the follow-up period without being evaluated by CT, were also considered as progression events, having verified that death was disease-related. One 10 ml EDTA blood tube was collected for all patients at baseline (before therapy start). At the same time, the same amount of blood was collected from 24 age and sex-matched healthy controls. The experimental protocols outlined above were approved by the Ethical Committee of the Complexo Hospitalario Universitario of Santiago de Compostela (institutional code of approval: 2009/289). All methods were carried out in accordance with the approved guidelines. All participants signed an informed consent specifically approved for this study.

### CTC isolation and gene expression analysis

Sample processing procedures have been previously described^52^. Briefly, CTCs were enriched from 7.5 ml of whole blood using anti-EpCAM coated magnetic beads (CELLection epithelial enrich, Life Technologies) and EpCAM^+^ isolated cells were pooled together from each patient. RNA was extracted with a methodology optimized for low concentration samples (Qiamp Viral, Qiagen) and cDNA was synthesized using SuperScriptIII polymerase (Life Technologies). To optimize target detection, samples were first preamplified (PreAmp Master Mix kit, Life Technologies). mRNA levels of CD45, CCDC88A, CCDC88C, NUCB1, NUCB1, S100A4 and MACC1 genes were quantified by quantitative Real-Time PCR using hydrolysis probes chemistry (Life Technologies) in a StepOne plus thermocycler (Life Technologies). Probe characteristics are detailed in Supplementary Table S2. Each sample was run in duplicate for each gene and appropriate negative controls were included in each qPCR reaction plate. Cq values (defined as the cycle number at which the fluorescence reached a fixed threshold value) for each transcript were normalized to 40 (maximum number of cycles), and this value to the 40-Cq value for CD45 (40-CqCD45), used as a reference gene as it detects hematopoietic cells unspecifically isolated.

### Statistical analysis

OS and PFS were defined as the time from start of treatment to death, or to the earlier of disease progression or death, respectively. Marker levels were classified as high or low when they were above or below the 75% percentile in these 51 patients. Kaplan-Meier (KM) curves and COX proportional hazards regression were used to study associations between marker levels and PFS/OS. Likelihood ratio tests were used to compare nested models. Cox models were evaluated using Harrell’s concordance index (c-index) and compared using the dependent sample t-test. Differences in gene expression between controls and patients, were analyzed using Mann-Whitney non-parametric test. Tests were performed with SPSSv20.0, GraphPad prism v5 or R v3.1.3 software, at the 5% significance level. AUCs were computed using GraphPad prism v5.

## Additional Information

**How to cite this article**: Barbazan, J. *et al.* Prognostic Impact of Modulators of G proteins in Circulating Tumor Cells from Patients with Metastatic Colorectal Cancer. *Sci. Rep.* **6**, 22112; doi: 10.1038/srep22112 (2016).

## Supplementary Material

Supplementary Information

## Figures and Tables

**Figure 1:**
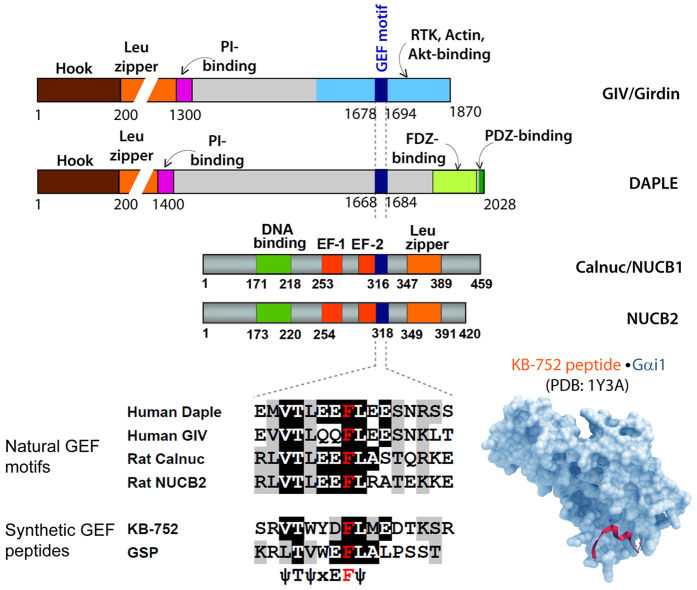
Domain composition of members of a new family of modulators of G protein that share an evolutionary conserved GEF motif as a common functional domain. Upper part of the figure displays bar diagrams drawn to scale showing the various functional domains of GIV, Daple, Calnuc/NUCB1 and NUCB2 (from top to bottom). The single domain that is shared by them all is the GEF motif shown in dark blue. The alignment of the sequences of GEF motifs with the sequences in synthetic peptides with demonstrated GEF activity towards Giα proteins is shown (lower left). The structural basis for interaction with Giα proteins has been solved for the synthetic KB-752 peptide (lower right), and homology modeling and extensive mutational analyses have confirmed that each member of the GEF family share these structural basis and molecular mechanisms of interaction with Giα.

**Table 1:** Genes studied in this work and their link to cancer progression.

Gene	Protein	Functional classification	Role in Cancer	Types of Cancer
CCDC88A	Gα-interacting vesicle-associated (GIV) protein (a.k.a. Girdin/HkRP1, APE).	Cytoskeleton associated guanine nucleotide exchange factor for trimeric G protein, Giα that modulates growth factor signaling.	Prometastatic (increases invasion, imparts chemoresistance, stem-ness, angiogenesis, survival). See Table S1.	Glioma, Esophagus, Gastric, Lung, Liver, Breast, Colon and Prostate.
CCDC88C	Dvl-associating protein with a high frequency of leucine residues (Daple)	Guanine nucleotide exchange factor for trimeric G protein, Giα that enhances non-canonical Wnt signaling.	Tumor suppressor in the normal epithelium; Proinvasive role in cancer cells. See Table S1.	Colon, Gastric
NUCB1	Nucleobindin1/Calnuc	EF-hand containing calcium binding protein and a guanine nucleotide exchange factor for trimeric G protein, Giα that is required for unfolded protein response. The role of its GEF function remains unknown.	Possible role in survival via regulation of UPR. See Table S1	Colon, Gastric
NUCB2	Nucleobindin2/Nesfatin-1/NEFA		Increases migration, proliferation and invasion. See Table S1.	Gastric, Prostate, Breast
MACC1	Metastasis Associated In Colon Cancer 1^17^	SH3 domain-containing protein that regulates HGF/c-Met pathway^17^	Increases migration, invasion, proliferation, survival and angiogenesis 53,54,55,56,57,58,59,60,61,62,63,64	Glioma, Esophagus, Lung Gastric, Liver, Breast, Colon Prostate, Klatskin, Salivary gland, Nasopharygeal and oral (tongue), Ovary, Cervix
S100A4	S100 Calcium Binding Protein A4 (a.k.a Calcium Protein, Calvasculin, Metastasin, Murine Placental Homolog)	EF-hand containing calcium binding protein that is member of the S100 calcium-binding protein family secreted by tumor and stromal cells	Supports tumorigenesis by stimulating angiogenesis.	Colon, Bladder, Lung, Breast, Prostate, Thyroid, Esophageal, Gallbladder, Gastric.

**Table 2:** Patient clinical characteristics.

Age (years)		
Mean	64,2	
SD	10,11	
Range	31–84	
Gender	***N***	**%**
Male	38	74,5
Female	13	25,5
Primary tumor location		
Colon	36	70,6
Rectum	13	25,5
Both	2	3,9
KRAS status		
Wild Type	32	62,7
Mutated	18	35,3
Unknown	1	2,0
T		
T_1_-T_2_	1	2,0
T_3_	37	72,5
T_4_	10	19,6
T_X_	3	5,9
N		
N_0_	4	7,8
N_1_	21	41,2
N_2_	21	41,2
N_X_	5	9,8
Number of metastatic sites		
1	24	47,1
2	21	41,2
≥3	6	11,7
Metastasis location		
Liver	19	37,3
Liver and other	25	49,0
Non liver	7	13,7
ECOG PS grade		
0	3	5,9
1	39	76,5
2	9	17,6
First line chemotherapy		
Folfox	42	82,4
Folfiri	3	5,9
Capecitabine	1	1,9
Capecitabine-Oxaliplatin	4	7,9
Irinotecan	1	1,9
First line combined biological therapy		
Anti-EGFR	19	37,2
Anti-VEGF	8	15,7
None	24	47,1

Abbreviations: SD; Standard deviation; ECOG, Eastern Cooperative Oncology Group; PS, Performance Status.

**Table 3:** Kaplan-Meier survival analysis for individual CTC markers.

		PFS (months)			OS (months)		
		Marker levels*		Median	95% CI	*P value*	Median	95% CI	*P value*
CCDC88A	Low	10,3	8,9–11,8	**0,007**	23,3	16,8–29,9	0,359
	High	7,0	2,0–11,0		9,3	7,5–11,1	
CCDC88C	Low	10,0	8,8–11,2	**0,003**	24,2	20,5–27,8	**0,003**
	High	5,6	3,2–8,0		7,1	4,2–10,0	
CCDC88Cfl	Low	10,0	8,8–11,2	**0,003**	23,3	19,9–26,8	**0,047**
	High	5,6	2,3–8,9		8,9	5,1–12,7	
NUCB1	Low	10,6	9,5–11,6	**<0,001**	24,1	20,5–27,8	**0,001**
	High	5,2	3,6–6,8		8,7	5,7–11,7	
NUCB2	Low	10,3	9,1–11,6	**0,005**	23,3	19,8–26,8	0,091
	High	6,6	0,9–12,3		9,3	6,8–11,9	
MACC1	Low	10,0	8,9–11,0	0,094	23,2	17,1–29,3	0,101
	High	6,6	3,8–9,4		7,0	2,3–11,6	
S100A4	Low	10,0	8,6–11,4	**0,033**	23,2	17,9–28,5	0,300
	High	8,5	2,6–14,4		10,4	5,4–15,3	

Abbreviations: CTC, Circulating Tumor Cells; PFS, Progression Free Survival; OS, Overall Survival;

CI, Confidence Interval. Times in months.

*Marker high and low levels were calculated based on 75% percentile for each marker.

**Table 4:** Univariate Cox proportional hazard regression analysis.

Covariate	*N*	PFS		OS	
		HR (95% CI)	*P value*	HR (95% CI)	*P value*
Age (<65 *vs* ≥65 years)	51	1,28 (0,70–2,35)	0,409	0,79 (0,40–1,57)	0,505
Sex (male *vs* female)	51	1,24 (0,44–2,06)	0,553	2,03 (0,83–4,96)	0,117
T stage (4 *vs* ≤3)	48	0,95 (0,38–1,82)	0,653	1,01 (0,43–2,37)	0,970
N stage (2 *vs* ≤1)	46	0,86 (0,45–1,62)	0,636	1,46 (0,69–3,04)	0,319
Hepatic mets. (yes *vs* no)	51	1,45 (0,64–3,30)	0,368	1,26 (0,52–3,05)	0,604
Lung mets. (yes *vs* no)	51	**3,11** (1,60–6,04)	**0,001**	**2,19** (1,09–4,39)	**0,027**
Peritoneal mets. (yes *vs* no)	51	1,16 (0,53–2,51)	0,713	1,39 (0,62–3,16)	0,424
N° of met sites (>2 *vs* ≤2)	51	2,12 (0,88–5,08)	0,094	**6,94** (2,48–19,37)	**<0,001**
KRAS (mut. *vs* WT)	50	0,97 (0,71–1,34)	0,868	0,82 (0,58–1,17)	0,274
Baseline CEA (≥75 ng/ml *vs* <75 ng/ml)	48	0,94 (0,51–1,73)	0,837	1,43 (0,71–2,86)	0,315
ECOG PS (2 *vs* ≤1)	51	1,72 (0,81–3,64)	0,155	**2,44** (1,13–5,26)	**0,023**
*CCDC88A*	51	**2,51** (1,25–4,99)	**0,009**	1,41 (0,67–2,96)	0,361
*CCDC88C*	51	**2,74** (1,38–5,45)	**0,004**	**2,88** (1,39–5,97)	**0,004**
*CCDC88Cfl*	51	**2,72** (1,37–5,42)	**0,004**	2,08 (1,00–4,33)	0,051
*NUCB1*	51	**3,62** (1,79–7,30)	**<0,001**	**3,01** (1,49–6,42)	**0,002**
*NUCB2*	51	**2,59** (1,29–5,17)	**0,007**	1,86 (0,89–3,87)	0,095
*MACC1*	51	1,79 (0,89–3,58)	0,099	1,83 (0,88–3,83)	0,105
*S100A4*	51	**2,07** (1,04–4,09)	**0,037**	1,48 (0,70–3,12)	0,303

Abbreviations: PFS, Progression Free Survival; OS, Overall Survival; HR, Hazard Ratio; CI, Confidence Interval; CEA, Carcinoembrionic antigen; ECOG, Eastern Cooperative Oncology Group; PS, Performance Status.

**Table 5:** Multivariate Cox proportional hazard regression analysis.

Covariates	*N*	PFS		χ^2^(**)	OS		χ^2^(**)
		HR (95% CI)	*P value*		HR (95% CI)	*P value*	
Number of metastatic locations (1,2 *vs* other)	51	0,72 (0,26–1,99)	0,524	**<0,001**	4,38 (1,35–14,20)	**0,014**	**<0,001**
Lung mets. (yes *vs* no)		3,75 (1,73–8,15)	**0,001**		1,85 (0,84–4,01)	0,125	
ECOG PS (2 *vs* ≤1)		1,790,75–4,29)	0,191		2,88 (1,22–6,78)	**0,016**	
GEF marker cluster		5,20 (2,15–12,57)	**<0,001**		2,69 (1,09–6,63)	**0,031**	
S100A4/MACC1 cluster		0,93 (0,43–2,00)	0,857		0,91 (0,40–2,07)	0,822	

Abbreviations: PFS, Progression Free Survival; OS, Overall Survival; HR, Hazard Ratio; CI, Confidence Interval; ECOG PS, Eastern Cooperative Oncology Group performance score.

(**) Significance of a Chi-square test for multivariate models.
