# Supplementary material for: Prognostic Impact of Modulators of G proteins in Circulating Tumor Cells from Patients with Metastatic Colorectal Cancer
Source: Sci Rep. 2016 Feb 26;6:22112. doi: 10.1038/srep22112 (PMC4768264; doi:10.1038/srep22112)
Supplement: Supplementary Information [file srep22112-s1.doc]

**Supplementary Information**

**Prognostic Impact of Modulators of G proteins in Circulating Tumor Cells from Patients with Metastatic Colorectal Cancer**

**Jorge Barbazan***^1¶^***, Ying Dunkel***^2¶^***, Hongying Li***^4^***, Ulrich Nitsche***^3^***, Klaus-Peter Janssen***^3^***, Karen Messer***^4^* **and Pradipta Ghosh***^2,4^,* ^*^

*^1^Translational Laboratory, Medical Oncology Department, Complexo Hospitalario Universitario de Santiago de Compostela/SERGAS, Santiago de Compostela, Spain.*

*^2^ Department of Medicine, University of California, San Diego, La Jolla, California, USA.*

*^3^ Department of Surgery, Klinikum rechts der Isar, TU München, Munich, Germany.*

*^4^ Division of Biostatistics, Department of Family Medicine and Public Health, Moores Cancer Center, University of California, San Diego, La Jolla, California, USA.*

**Supplementary tables.**

Table S1

| **Supplementary Table S1: Significance of Non-Receptor GEFs in Cancer Progression** | | | | |
| --- | --- | --- | --- | --- |
| **Patho-**  **physiologic**  **Processes** | **Approach [model system(s)] and**  **Parameter(s) analyzed)** | **Effect of the GEF function** | **Receptor(s) studied** | **Ref** |
| **Role of GIV in Cancer Progression** | **Parameters:**  **Migration** and **Invasion**.  **Approach:** Gene depletion studies in multiple cancer cell lines for use in matrigel invasion; haptotaxis, haptoinvasion assays. Distant metastasis in murine models of cancer dissemination. | "ON" = Enhances  "OFF" = Inhibits | IGF1R, EGFR, integrin β1, Multi-receptor* | 1-11 |
|  | **Parameters**: **Stemness.**  **Approach:** CD133, multilineage neural differentiation, in vitro cell motility, ex vivo invasion, sphere-formation, in vivo tumor formation were analyzed in control and GIV-depleted glioblastoma cells. | Not examined | -- | 12 |
|  | **Parameter:** **Chemoresistance.**  **Approach:** Survival was assessed in control and GIV-depleted colorectal cancer cells exposed to Oxaliplatin and Adriamycin using MTT assays. | Not examined | -- | 13 |
|  | **Parameter:** **Tumor-Stroma Interactions.**  **Approach:** Assessment of distant metastasis after grafting Lewis lung cancer cells in GIV transgenic mice (knock-in of a defective mutant). | Not examined | PDGFR, TGFβR, CXCR4 | 14 |
|  | **Parameters**: **Angiogenesis and** **Neovascularization**  **Approach**: siRNA implantation in matrigel; knockout mice were used to study VEGF triggered angiogenesis in mice subcutaneous implants; retinal and aortic ring vasculature in knockout mice. | Not examined | VEGFR | 15 |
|  | **Parameters: Prognostic significance of GIV across a variety of solid tumors**  **Approach:** Multiple studies, on patient samples, using IHC as the primary approach.  All studies agree, in general, that high levels of GIV equals more aggressive tumors. | Not examined | -- | Summarized in ^16^ |
|  | **Parameter: Mechanisms of deregulation during oncogenesis**  Expression of GIV is decreased initially, via alternative splicing, and then increased later during cancer progression ^17^. Transcriptional upregulation during cancer progression is mediated by STAT3 ^2^. |  |  |  |
| **Role of Daple in Cancer Progression** | **Parameters: Cell motility and migration**  **Approach**: Gene depletion studies and expression of WT vs mutant Daple in cancer cells for use in matrigel invasion, 2D migration, and chemotactic migration using Transwell® assays. Mouse knockout model used to study Daple's role during cell migration after dermal wounding; measurement of Akt and Rac1 signals. | "ON" = enhances  "OFF" = inhibits | Wnt5a/Fzld | 7 |
|  | **Parameters:** **Tumor cell proliferation and growth**  **Approach:**  Gene depletion studies and expression of WT vs mutant Daple in cancer cells for use in anchorage-dependent and independent colony formation assays; measurement of β-Catenin/TCF/LEF signals. | "ON" = inhibits  "OFF" = triggers | Wnt5a/Fzld | 7 |
|  | **Parameter:** **Prognostic significance of Daple in colorectal cancer**  **Approach:**  Analysis of Daple in patient-derived normal and cancer tissue, and in circulating tumor cells by qPCR. | -- | -- | 7 |
|  | **Parameter: Mechanisms of deregulation during oncogenesis**  Reduction in DNA copy number during polyp-to-cancer progression. Increased expression in invasive front of primary tumor and circulating tumor cells via unknown mechanism^7^. Chromosomal translocations resulting in gene fusions described in leukemias and lymphomas ^18^. | -- | -- | 7,18 |
| **Role of Calnuc/NUCB1 and NUCB2 in Cancer Progression** | **Parameters: Cancer cell profileration, migration and invasion**  **Approach**: Gene depletion studies confirmed that NUCB2 significantly increased cell proliferation, and migration and invasion properties of breast cancer cells. | Not examined | -- | 19 |
|  | **Parameter:** **Prognostic and diagnostic significance of NUCB1/NUCB2 in cancer**  **Approach:**  Analysis of NUCB2 in patient-derived normal and cancer tissue by IHC ^20,21^; analysis of NUCB2 mRNA by qPCR ^22^. Autoantibodies in patients with gastric cancer^23^. The studies agree that high expression equals poorer outcome. | -- | -- | 20-25 |
|  | **Parameter: Mechanisms of deregulation during oncogenesis**  Increased expression in response to Estrogens^19^, possible transcriptional activation by ER-stress and ATF6^26^; mRNA stabilization in response to PPARγ agonists^27^. | -- | -- |  |

**Table S2**

| **Supplementary Table S2: qPCR probes** | | | |
| --- | --- | --- | --- |
| **Gene Name** | **Probe number** | **RefSeq(s)** | **Amplicon length (BP)** |
| CD45 | Hs00894734_m1 | NM_002838.3 | 70 |
| GAPDH | Hs99999905_m1 | NM_002046.3 | 122 |
| RPLPO | Hs99999902_m1 | NM_001002.3 | 105 |
|  |  | NM_053275.3 |  |
| ACTB | Hs99999903_m1 | NM_001101.3 | 171 |
| CCDC88A | Hs01554973_m1 | NM_001135597.1 | 130 |
|  |  | NM_001254943.1 |  |
|  |  | NM_018084.4 |  |
| CCDC88C | Hs00325884_m1 | NM_001080414.3 | 100 |
| CCDC88Cfl | Hs00380245_m1 | NM_001080414.3 | 78 |
| NUCB1 | Hs00939167_m1 | NM_006184.5 | 96 |
| NUCB2 | Hs01093819_m1 | NM_005013.2 | 91 |
| S100A4 | Hs00243202_m1 | NM_002961.2 | 101 |
|  |  | NM_019554.2 |  |
| MACC1 | Hs00766186_m1 | NM_182762.3 | 113 |
| Abbreviations: BP, Base pairs. | | | |

**Table S3**

**
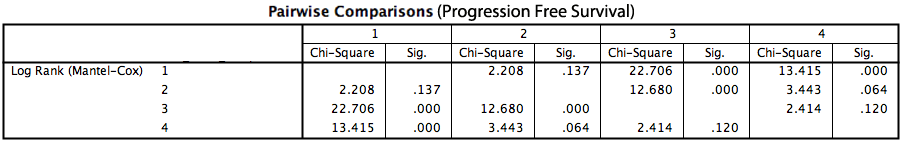

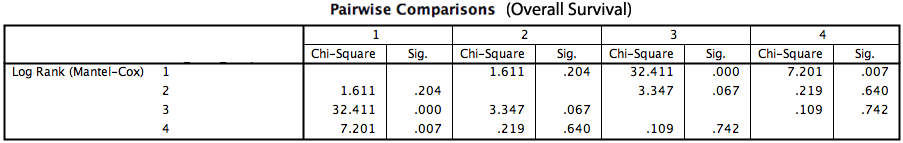
**

**REFERENCES**

1 Bhandari, D. *et al.* Cyclin-dependent kinase 5 activates guanine nucleotide exchange factor GIV/Girdin to orchestrate migration-proliferation dichotomy. *Proc Natl Acad Sci U S A*, doi:10.1073/pnas.1514157112 (2015).

2 Dunkel, Y. *et al.* STAT3 protein up-regulates Galpha-interacting vesicle-associated protein (GIV)/Girdin expression, and GIV enhances STAT3 activation in a positive feedback loop during wound healing and tumor invasion/metastasis. *J Biol Chem* **287**, 41667-41683, doi:10.1074/jbc.M112.390781 (2012).

3 Enomoto, A. *et al.* Akt/PKB regulates actin organization and cell motility via Girdin/APE. *Dev Cell* **9**, 389-402, doi:10.1016/j.devcel.2005.08.001 (2005).

4 Jiang, P. *et al.* An actin-binding protein Girdin regulates the motility of breast cancer cells. *Cancer Res* **68**, 1310-1318, doi:10.1158/0008-5472.CAN-07-5111 (2008).

5 Leyme, A., Marivin, A., Perez-Gutierrez, L., Nguyen, L.T. and Garcia-Marcos, M. . Integrins activate trimeric G proteins via the non-receptor protein GIV/ Girdin *J Cell Biol* (2015 (Accepted, In Press)).

6 Lopez-Sanchez, I., Kalogriopoulos, N., Lo, I., Kabir, F., Midde, K., Wang H. and Ghosh, P. Focal Adhesions are Foci for Tyrosine-Based Signal Transduction via GIV/Girdin and G proteins. *Mol Biol Cell* **Accepted, In Press.** (2015).

7 Aznar, N. *et al.* Daple is a novel non-receptor GEF required for trimeric G protein activation in Wnt signaling. *Elife* **4**, e07091, doi:10.7554/eLife.07091 (2015).

8 Midde, K. K. *et al.* Multimodular biosensors reveal a novel platform for activation of G proteins by growth factor receptors. *Proc Natl Acad Sci U S A* **112**, E937-946, doi:10.1073/pnas.1420140112 (2015).

9 Ohara, K. *et al.* Involvement of Girdin in the determination of cell polarity during cell migration. *PLoS One* **7**, e36681, doi:10.1371/journal.pone.0036681 (2012).

10 Wang, C., Lin, J., Li, L. & Wang, Y. Expression and clinical significance of girdin in gastric cancer. *Mol Clin Oncol* **2**, 425-428, doi:10.3892/mco.2014.265 (2014).

11 Wang, Y. *et al.* A chimeric antibody targeting CD147 inhibits hepatocellular carcinoma cell motility via FAK-PI3K-Akt-Girdin signaling pathway. *Clin Exp Metastasis* **32**, 39-53, doi:10.1007/s10585-014-9689-7 (2015).

12 Natsume, A. *et al.* Girdin maintains the stemness of glioblastoma stem cells. *Oncogene* **31**, 2715-2724, doi:10.1038/onc.2011.466 (2012).

13 Zhang, Y. J., Li, A. J., Han, Y., Yin, L. & Lin, M. B. Inhibition of Girdin enhances chemosensitivity of colorectal cancer cells to oxaliplatin. *World J Gastroenterol* **20**, 8229-8236, doi:10.3748/wjg.v20.i25.8229 (2014).

14 Yamamura, Y. *et al.* Akt-Girdin signaling in cancer-associated fibroblasts contributes to tumor progression. *Cancer Res* **75**, 813-823, doi:10.1158/0008-5472.CAN-14-1317 (2015).

15 Kitamura, T. *et al.* Regulation of VEGF-mediated angiogenesis by the Akt/PKB substrate Girdin. *Nat Cell Biol* **10**, 329-337, doi:10.1038/ncb1695 (2008).

16 Garcia-Marcos, M., Ghosh, P. & Farquhar, M. G. GIV/Girdin transmits signals from multiple receptors by triggering trimeric G protein activation. *J Biol Chem* **290**, 6697-6704, doi:10.1074/jbc.R114.613414 (2015).

17 Ghosh, P. *et al.* A G{alpha}i-GIV molecular complex binds epidermal growth factor receptor and determines whether cells migrate or proliferate. *Mol Biol Cell* **21**, 2338-2354, doi:10.1091/mbc.E10-01-0028 (2010).

18 Gosenca, D. *et al.* Identification and functional characterization of imatinib-sensitive DTD1-PDGFRB and CCDC88C-PDGFRB fusion genes in eosinophilia-associated myeloid/lymphoid neoplasms. *Genes Chromosomes Cancer* **53**, 411-421 (2014).

19 Suzuki, S. *et al.* Nucleobindin 2 in human breast carcinoma as a potent prognostic factor. *Cancer Sci* **103**, 136-143, doi:10.1111/j.1349-7006.2011.02119.x (2012).

20 Qi, C., Ma, H., Zhang, H. T., Gao, J. D. & Xu, Y. Nucleobindin 2 expression is an independent prognostic factor for clear cell renal cell carcinoma. *Histopathology* **66**, 650-657, doi:10.1111/his.12587 (2015).

21 Wang, S. N. *et al.* Antigen expression associated with lymph node metastasis in gastric adenocarcinomas. *Pathol Int* **44**, 844-849 (1994).

22 Zhang, H., Qi, C., Li, L., Luo, F. & Xu, Y. Clinical significance of NUCB2 mRNA expression in prostate cancer. *J Exp Clin Cancer Res* **32**, 56, doi:10.1186/1756-9966-32-56 (2013).

23 Chen, Y. *et al.* Autoantibodies to Ca2+ binding protein Calnuc is a potential marker in colon cancer detection. *Int J Oncol* **30**, 1137-1144 (2007).

24 Zhang, H., Qi, C., Wang, A., Li, L. & Xu, Y. High expression of nucleobindin 2 mRNA: an independent prognostic factor for overall survival of patients with prostate cancer. *Tumour Biol* **35**, 2025-2028, doi:10.1007/s13277-013-1268-z (2014).

25 Zhang, H. *et al.* Prognostication of prostate cancer based on NUCB2 protein assessment: NUCB2 in prostate cancer. *J Exp Clin Cancer Res* **32**, 77, doi:10.1186/1756-9966-32-77 (2013).

26 Tsukumo, Y. *et al.* Nucleobindin 1 controls the unfolded protein response by inhibiting ATF6 activation. *J Biol Chem* **282**, 29264-29272, doi:10.1074/jbc.M705038200 (2007).

27 Yamada, M. *et al.* Troglitazone, a ligand of peroxisome proliferator-activated receptor-{gamma}, stabilizes NUCB2 (Nesfatin) mRNA by activating the ERK1/2 pathway: isolation and characterization of the human NUCB2 gene. *Endocrinology* **151**, 2494-2503, doi:10.1210/en.2009-1169 (2010).
